# Repurposing Approved Drugs as Fluoroquinolone Potentiators to Overcome Efflux Pump Resistance in Staphylococcus aureus

**DOI:** 10.1128/Spectrum.00951-21

**Published:** 2021-12-15

**Authors:** Nisha Mahey, Rushikesh Tambat, Nishtha Chandal, Dipesh Kumar Verma, Krishan Gopal Thakur, Hemraj Nandanwar

**Affiliations:** a Clinical Microbiology & Antimicrobial Research Laboratory, CSIR-Institute of Microbial Technology, Chandigarh, India; b Academy of Scientific & Innovative Research (AcSIR), Ghaziabad, Uttar Pradesh, India; c Structural Biology Laboratory, CSIR-Institute of Microbial Technology, Chandigarh, India; Riverside University Health System, Medical Center, University of California

**Keywords:** *Staphylococcus aureus*, major facilitator superfamily (MFS), NorA efflux pump, efflux pump inhibitors, drug repurposing

## Abstract

Staphylococcus aureus is a versatile human commensal bacteria and pathogen that causes various community and hospital-acquired infections. The S. aureus efflux pump NorA which belongs to the major facilitator superfamily, confers resistance to a range of substrates. Many efflux pump inhibitors (EPIs) have been discovered, but none is clinically approved due to their undesirable toxicities. In this study, we have screened clinically approved drugs for possible NorA EPI-like activity. We identified six drugs that showed the best efflux pump inhibition *in vitro*, with a fractional inhibitory concentration index of ≤0.5, indicating synergism with hydrophilic fluoroquinolones. The mechanistic validation of efflux inhibitory potential was demonstrated in ethidium bromide-based accumulation and efflux inhibition assays. We further confirmed the functionality of EPIs by norfloxacin accumulation assay depicting more realistic proof of the conjecture. None of the EPIs disturbed membrane function or depleted the ATP synthesis levels in bacteria. Both raloxifene and pyrvinium displayed an increase in bactericidal activity of ciprofloxacin in time-kill kinetics, prolonged its post-antibiotic effect, and reduced the frequency of spontaneous resistant mutant development. The combination of EPIs with ciprofloxacin caused significant eradication of preformed biofilms. Moreover, in the murine thigh infection model, a single dose of pyrvinium combined with ciprofloxacin reduced the bacterial burden significantly compared to untreated control and ciprofloxacin alone, indicating the efficacy of the combination. Conclusively, this study represents approved drugs that can be repurposed and combined with antibiotics as NorA EPIs, having anti-biofilm properties to treat severe S. aureus infections at clinically relevant concentrations.

**IMPORTANCE** Staphylococcus aureus is a frequent pathogen bacterium and the predominant cause of worsened nosocomial infections. Efflux pumps contribute to drug efflux and are reportedly associated with biofilm formation, thereby promoting difficult-to-treat biofilm-associated S. aureus infections. One strategy to combat these bacteria is to reduce active efflux and increase pathogen sensitivity to existing antibiotics. Repurposing approved drugs may solve the classical toxicity issues with previous efflux pump inhibitors and help reach sufficient plasma concentrations. We describe the *in silico*-based screening of FDA-approved drugs that identified six different molecules able to inhibit NorA pump (Major Facilitator Superfamily). Our study highlights that these compounds bind to and block the activity of the NorA pump and increase the sensitivity of S. aureus and methicillin-resistant S. aureus to fluoroquinolones. These drugs combined with fluoroquinolones significantly reduced the preformed biofilms and displayed significant efficacy in the murine thigh infection model when compared to untreated control and ciprofloxacin alone.

## INTRODUCTION

Staphylococcus aureus is a frequent human commensal bacterium and the foremost cause of bacteremia, endocarditis, osteomyelitis, and skin and soft tissue infections. It has become a prime cause of steadily increasing health care-associated infections imposing an elevated burden on health care resources. Moreover, Methicillin-resistant Staphylococcus aureus (MRSA) accounts for 12% of healthcare-associated infections (HAIs) in the adult population and is among the three most frequently reported pathogens contributing to HAIs, according to a report of the National Healthcare Safety Network (1). The pathogenic potential of S. aureus is not only limited to planktonic bacteria but also extends to biofilms. Progressively, it has developed the ability to promptly develop resistance to any antibiotic by using various resistance mechanisms, including enzymatic inactivation of the antibiotic, alteration of the target, trapping of the antibiotic, and efflux pumps (2).

Contrary to other resistance mechanisms, efflux-mediated resistance has recently gathered more interest in S. aureus, as many bacterial efflux pumps can expel several unrelated classes of antimicrobials, thereby promoting multi-drug resistance. The major facilitator superfamily (MFS) efflux pumps are abundant and the most diverse family of transporters present in S. aureus (3). The MFS superfamily’s members are predominantly monomeric proteins with lengths ranging from 388 to 600 amino acids. They possess 12–14 transmembrane helices that form two domains, and each domain is composed of bundles of six helices. It includes uniporters (transport of substrates without coupling ions across the bilayer) and symporters (the unidirectional coupled substrate-ion transport across the bilayer) (3). NorA in S. aureus, which is chromosomally coded by the *norA* gene, is one of the most researched MFS efflux pumps. NorA is a protein comprising 388 amino acids, 12 transmembrane segments and transports the antimicrobial agents across the cell membrane using the proton motive force (drug/H^+^ antiport) (4). It can extrude various chemically and structurally different compounds, such as dyes like ethidium bromide (EtBr), hydrophilic fluoroquinolones, i.e., norfloxacin and ciprofloxacin, and biocides such as quaternary ammonium compounds. The clinical significance of NorA efflux pumps can be estimated by a study indicating a high prevalence of *norA* gene expression in bloodstream S. aureus isolates (5). Some earlier studies have reported the over-expression of *norA* efflux pump gene in the fluoroquinolone-resistant clinical isolates (6, 7).

Efflux pump inhibitors (EPIs) work as potentiators of existing antibiotics and help in a reversal of intrinsic and acquired resistance in bacteria (8, 9). Many EPIs have been discovered in the past years, e.g., reserpine, but none is clinically approved due to their unavoidable toxicities (10, 11).

The rate of spread of resistance is swift, which is further assisted by the dried antibiotic pipeline. A possible strategy is to evaluate the efficacy of previously approved antibiotics. The previously approved drugs can be explored as potentiators with known antibiotics. The already assessed pharmacokinetics, pharmacodynamics, and dosing range make the drugs’ repurposing beneficial. Considering the clinical significance of NorA efflux pump, a NorA inhibitor-antibiotic combination therapy could be a promising alternative to tackle multi-drug resistance.

In this study, a Food and Drug Administration (FDA) approved small molecule drugs library was screened for possible inhibitory potential against the NorA pump. We shortlisted six EPI-like drugs through virtual screening followed by *in vitro* antibiotic potentiation assays. This study also demonstrates the remarkable potency of our best EPI, i.e., pyrvinium combined with ciprofloxacin in a murine thigh infection model. To our knowledge, this is the first description of an approved drug that can be used as an EPI *in vivo* and to reduce biofilm formation at clinically achievable concentrations.

## RESULTS

### Virtual screening and molecular docking to the active site of NorA.

**FIG 1:**
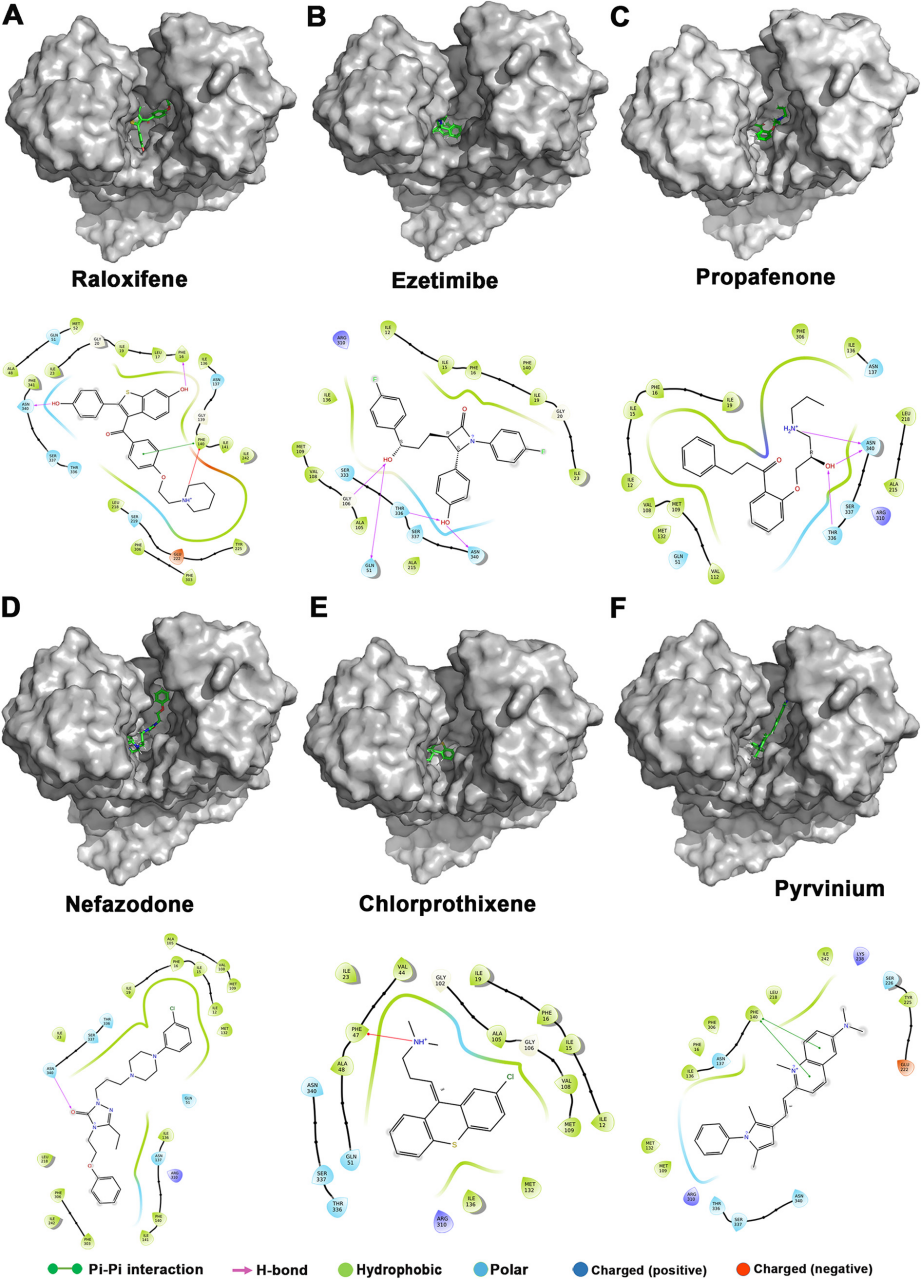
Interaction of (A) raloxifene, (B) ezetimibe, (C) propafenone, (D) nefazodone, (E) chlorprothixene, and (F) pyrvinium with the active site of NorA. 3D electrostatic potential map (upper panel) showing EPIs docked in the active site cleft. Ligand interaction diagram in 2D representation showing several interactions involved in NorA-EPI binding (lower panel).

The FDA-approved drug library (1,965 drugs) was docked against the entire surface of the NorA homology model, and the free energies, as well as the Molecular Mechanics energies combined with the Generalized Born and Surface Area continuum solvation (MM-GBSA) scores of the best binding poses, were calculated (Table S1). The molecular modeling studies revealed multiple FDA-approved drugs as potential binders with high binding scores. The initial virtual screening using *in silico* molecular docking followed by *in vitro* screening (∼300 compounds) utilized in this study identified six potential hits, i.e., raloxifene, ezetimibe, propafenone, nefazodone, chlorprothixene, and pyrvinium. Among the compounds, raloxifene showed the highest docking and MM-GBSA score of –9.064 kcal/mol and –70.08 kcal/mol, respectively, while chlorprothixene exhibited the lowest docking score of –3.757 kcal/mol. Based on MM-GBSA based binding energy calculations, all six compounds have NorA binding potential in the order of raloxifene > nefazodone > ezetimibe > chlorprothixene > propafenone > pyrvinium. The moderate to high docking and MMGBSA scores suggest that these drugs may have favorable interactions and inhibit NorA activity. The important NorA interacting residues are Asn340, Phe16, Gln51, Thr336, and Phe140, forming hydrogen bonds and pi-pi interactions with the compounds and stabilizing their interaction with the binding core (Fig. 1).

### The compounds do not influence the natural growth kinetics of bacteria.

Bacterial growth kinetics is an autocatalytic reaction depicting direct proportionality between growth rate and the concentration of cells (12). We have assessed the effect of 1/4 × MICs of compounds, i.e., raloxifene, ezetimibe, propafenone, nefazodone, chlorprothixene, and pyrvinium on primitive bacterial growth kinetics of S. aureus SA-1199B. Ideally, they should not alter typical bacterial growth patterns at sub-inhibitory concentrations. None of the compounds affected the typical growth pattern of bacteria; no difference was observed compared to the control group (Fig. S1).

### Repurposed drugs reverse fluoroquinolones resistance phenotype.

**FIG 2:**
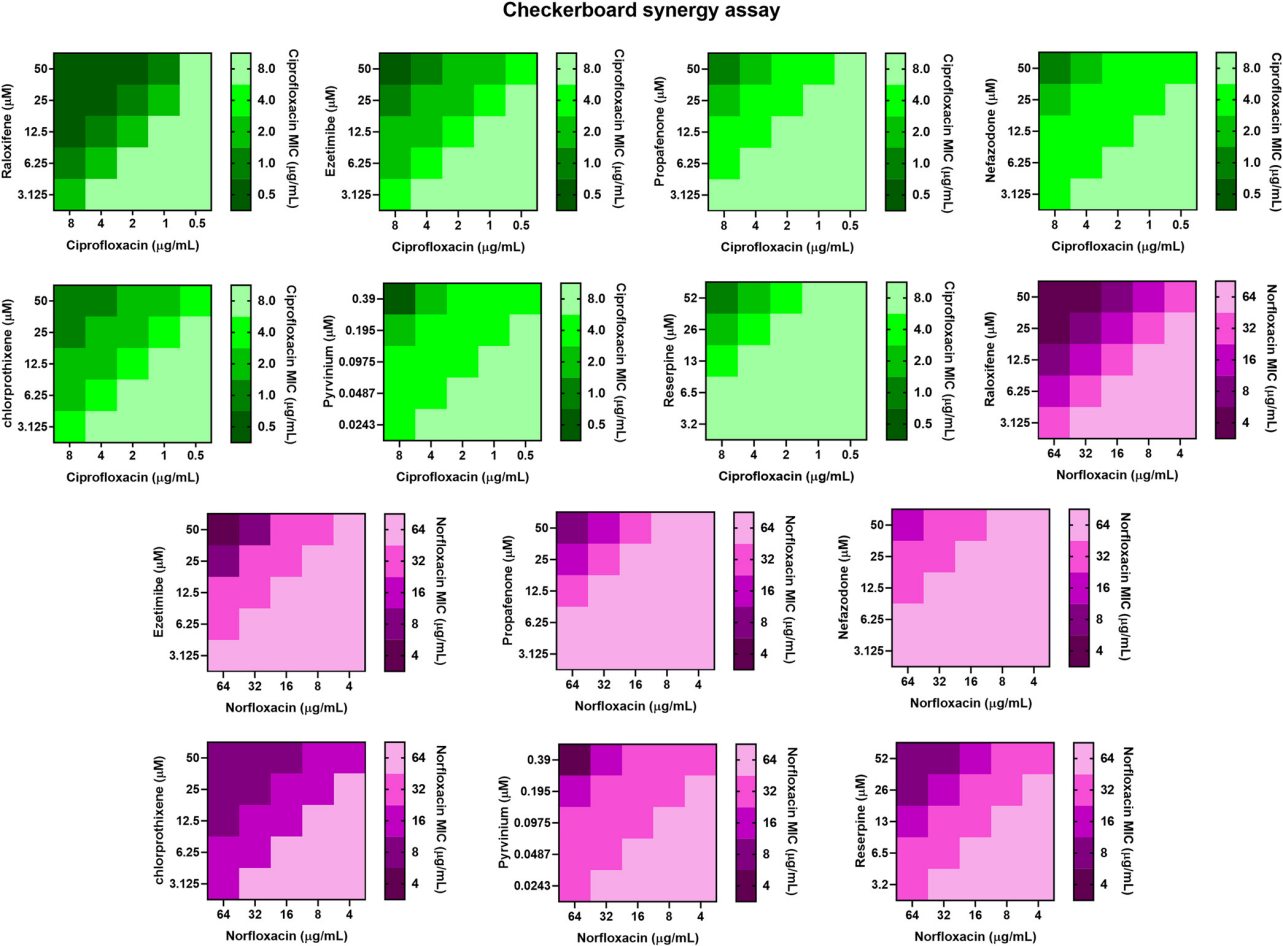
Heat-maps representing the synergistic activity of raloxifene, ezetimibe, propafenone, nefazodone, chlorprothixene, pyrvinium, and reserpine in combination with ciprofloxacin (gradients of green) and norfloxacin (gradients of purple) against S. aureus SA-1199B.

Next, we performed a checkerboard synergy assay on S. aureus SA-1199B (*norA* over-expressed strain), S. aureus SA-1199 (wild-type), and S. aureus K1758 (*norA* deletion strain) to determine the synergistic activity of compounds in combination with ciprofloxacin and norfloxacin. The MICs of compounds, ciprofloxacin, and norfloxacin were determined against various strains used in the study. The results indicate that compounds at sub-inhibitory concentrations (1/4 × MICs), i.e., raloxifene (50 μM), ezetimibe (50 μM), propafenone (50 μM), nefazodone (50 μM), chlorprothixene (50 μM), and pyrvinium (0.39 μM) modulated the MIC of ciprofloxacin for S. aureus SA-1199B by 8–16-fold. Moreover, the same concentrations in combination with norfloxacin modulated the norfloxacin’s MIC by 4–16-fold (Table 1). The fractional inhibitory concentration indices (FICIs) of all compounds at 1/4 × MIC was ≤0.5, which indicates synergy. Most importantly, raloxifene, ezetimibe, and pyrvinium displayed better synergistic activity than reserpine (used as a positive control due to known NorA efflux inhibitory activity). The concentration-dependent potentiation effect was observed for all the compounds (Fig. 2). However, for S. aureus SA-1199 (wild-type), the compounds at sub-inhibitory concentrations, i.e., raloxifene (50 μM), ezetimibe (50 μM), propafenone (50 μM), nefazodone (50 μM), chlorprothixene (50 μM), and pyrvinium (0.39 μM) modulated the MIC of ciprofloxacin by 2–4-fold, and the MIC of norfloxacin by 2–4-fold (Table S2). The relatively weak modulation effect is attributed to the basal level expression of NorA efflux pump in wild-type S. aureus (SA-1199). The synergism with antibiotics can be due to the NorA efflux pump inhibition; to confirm this conjecture, we also determined the synergism on the *norA* deletion strain (Table 1). None of the compounds displayed a significant reduction in the MIC of ciprofloxacin, and we can consider the compounds as NorA EPIs.

**TABLE 1:** MIC of ciprofloxacin, norfloxacin, drugs for S. aureus SA-1199B, K1758 alone, and MIC of antibiotics in combination with different drugs^*a*^

Compound	MIC of compound (μM)	Conc. used (μM)	MIC (μg/mL) in the presence of compound			Fractional inhibitory concn index (FICI)	
			S. aureus SA-1199B		S. aureus K1758	S. aureus SA-1199B	
			CIP	Nor	CIP	CIP	Nor
			8	64	0.125	NA	NA
Raloxifene	200	50	0.5	4	-	0.3125	0.3125
		25	0.5	4	-	0.1875	0.1875
		12.5	0.5	8	-	0.125	0.1875
		6.25	1	16	-	0.1562	0.28125
		3.125	2	32	-	0.2656	0.5156
Ezetimibe	200	50	0.5	4	-	0.3125	0.3125
		25	1	8	-	0.25	0.25
		12.5	2	32	-	0.3125	0.5625
		6.25	2	32	-	0.2812	0.5312
		3.125	4	64	-	0.5156	1.0156
Propafenone	800	50	1	8	-	0.1875	0.375
		25	2	16	-	0.2812	0.375
		12.5	4	32	-	0.5156	0.5625
		6.25	4	64	-	0.5078	1.0312
		3.125	8	64	-	1.003	1.0156
Nefazodone	200	50	1	16	-	0.375	0.5
		25	2	32	-	0.375	0.0625
		12.5	4	32	-	0.5625	0.5625
		6.25	4	64	-	0.5312	1.0312
		3.125	4	64	-	0.5156	1.0156
Chlorprothixene	200	50	1	8	0.0625	0.375	0.375
		25	1	8	-	0.25	0.25
		12.5	2	8	-	0.3125	0.1875
		6.25	2	16	-	0.2812	0.2812
		3.125	4	16	-	0.5156	0.2656
Pyrvinium	1.56	0.39	0.5	4	-	0.3125	0.3125
		0.195	2	16	-	0.375	0.375
		0.097	4	32	-	0.5625	0.05625
		0.048	4	32	-	0.5312	0.5312
		0.024	4	32	-	0.5156	0.5156
Reserpine	210	52	1	8	-	0.375	0.375
		26	2	8	-	0.375	0.25
		13	4	16	-	0.5625	0.3125
		6.5	8	32	-	1.0312	0.5312
		3.2	8	32	-	1.0156	0.5156

a CIP, ciprofloxacin; NOR, norfloxacin; NA, not applicable; “-”, no change in MIC.

**FIG 3:**
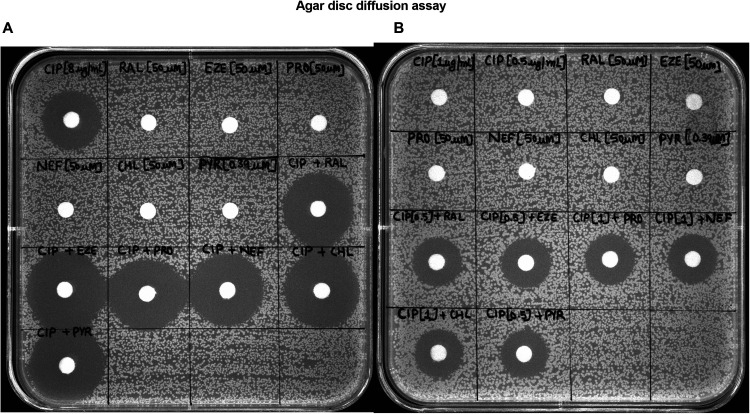
(A) Synergy test using disc-diffusion assay on S. aureus SA-1199B. The discs containing compounds (1/4 × MIC), ciprofloxacin (8 μg/mL) alone, and the combination of ciprofloxacin (8 μg/mL) with all the compounds. (B) The discs containing ciprofloxacin at the synergistic concentration (1 μg/mL, 0.5 μg/mL) (i.e., the reduced MIC of ciprofloxacin in the presence of drugs). The discs containing compounds (1/4 × MIC) combined with ciprofloxacin (1 μg/mL, 0.5 μg/mL). The images are representatives of two independent experiments. CIP, ciprofloxacin; RAL, raloxifene; EZE, ezetimibe; PRO, propafenone; NEF, nefazodone; CHL, chlorprothixene; PYR, pyrvinium.

For the reader-friendly visualization of the synergy, we designed an agar plate-based disc-diffusion assay. As expected, compounds alone (raloxifene, ezetimibe, propafenone, nefazodone, chlorprothixene, and pyrvinium) at sub-inhibitory concentrations (1/4 × MIC) did not show any zone of growth inhibition. The disc of ciprofloxacin alone produced a zone of growth inhibition of 20 mm in diameter. In the combination, the zone of growth inhibition increased up to ∼ 4–6 mm (Fig. 3A). Also, the discs with synergistic concentrations (concentrations of ciprofloxacin to which its MIC is reduced in the presence of compounds) of ciprofloxacin, i.e., 0.5 μg/ml and 1 μg/ml, did not display any growth inhibition zone. However, the combination of ciprofloxacin and compounds produced a zone of ∼16 mm for each combination set, which represents synergy (Fig. 3B).

Furthermore, the synergistic activity of the combination against clinical strains of MRSA was determined. The combination of all six compounds with ciprofl oxacin reduced its MIC by 2–32-fold, thereby increasing the susceptibility of bacteria to ciprofloxacin (Table 2). Moreover, we performed a checkerboard synergy assay with moxifloxacin against the strain over-expressing NorA (S. aureus SA-1199B) and against S. aureus clinical strains. NorA efflux pump extrudes a variety of substrates, including hydrophilic fluoroquinolones (ciprofloxacin, norfloxacin) except hydrophobic fluoroquinolones (moxifloxacin). Moxifloxacin is a known substrate of NorB efflux pump (13) and not NorA efflux pump. We observed that none of all six compounds exhibited synergy with moxifloxacin (Table S3, Table S4). Moreover, no synergism was observed in a strain expressing TetK (S. aureus XU212) when the compounds were combined with the antibiotic substrate of the efflux pump, i.e., tetracycline (Table S3). Altogether these results suggest that NorA is a target of the compounds in the study.

**TABLE 2:** MIC of ciprofloxacin (μg/ml) for S. aureus clinical strains (MRSA) in the presence of drugs/compounds^*a*^

Strain	Minimum inhibitory concn of ciprofloxacin (μg/mL)						
	−	+ Ral (50 μM)	+ Eze (50 μM)	+ Pro (50 μM)	+ Nef (50 μM)	+ Chl (50 μM)	+ Pyr (0.78 μM)
MRSA 1	16	1	1	2	2	4	2
MRSA 2	8	2	2	4	4	4	2
MRSA 3	16	2	4	4	4	4	4
MRSA 4	64	2	4	4	8	8	4
GMCH 831	32	2	4	4	8	8	4
GMCH 839	16	2	2	4	4	4	2

a Ral, raloxifene; Eze, ezetimibe; Pro, propafenone; Nef, nefazodone; Chl, chlorprothixene; Pyr, pyrvinium.

### The EPIs inhibited EtBr efflux.

**FIG 4:**
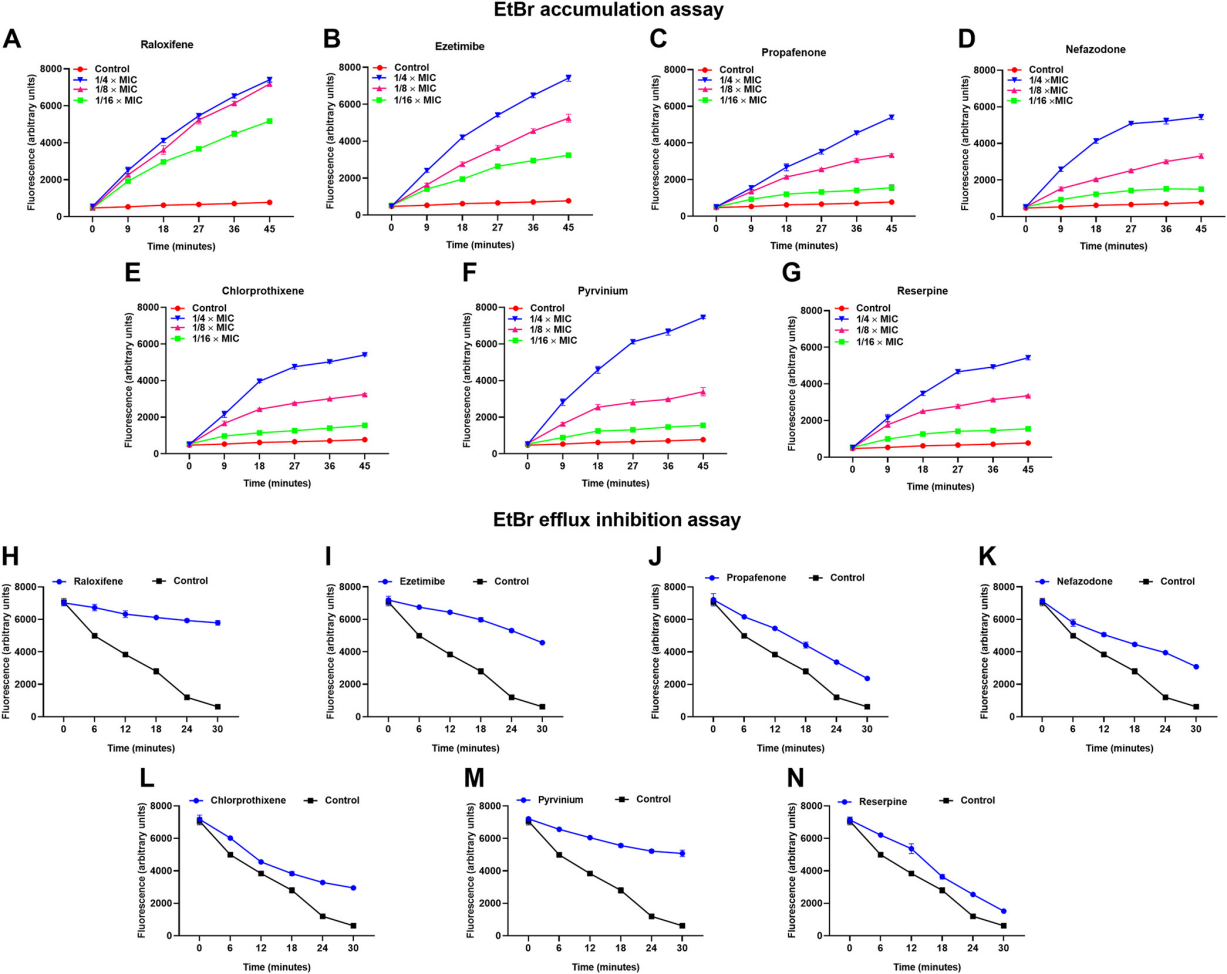
Effect of sub-inhibitory concentrations (1/4 × MIC) of (A) raloxifene, (B) ezetimibe, (C) propafenone, (D) nefazodone, (E) chlorprothixene, (F) pyrvinium on the real-time accumulation of EtBr in a concentration-dependent manner; the positive control (G) reserpine was included for comparison. The results presented correspond to the average of three independent assays ± SD. Effect of sub-inhibitory concentrations (1/4 × MIC) of (H) raloxifene, (I) ezetimibe, (J) propafenone, (K) nefazodone, (L) chlorprothixene, (M) pyrvinium on the efflux inhibition of EtBr; the positive control reserpine (N) was included for comparison. The results presented correspond to the average of three independent assays ± SD.

To further confirm that the synergism observed in the checkerboard synergy assay is due to the efflux inhibition, we performed the real-time EtBr accumulation assay on S. aureus SA-1199B. EtBr is a known substrate of several chromosomally encoded efflux pumps, which binds to DNA and fluoresce. We observed a concentration-dependent increase in EtBr accumulation compared to the control when treated with six hit-compounds individually (Fig. 4A to G). The increase in dye accumulation indicates the halted extrusion of dye due to NorA efflux pump inhibition.

Furthermore, the compounds’ potential to inhibit EtBr extrusion from S. aureus SA-1199B was determined. The increased expression of the NorA efflux pump caused rapid extrusion of EtBr, due to which a decrease in fluorescence was observed. However, the presence of sub-inhibitory concentrations (1/4 × MIC) of compounds (raloxifene, ezetimibe, propafenone, nefazodone, chlorprothixene, and pyrvinium) significantly slowed EtBr extrusion compared to the untreated control in S. aureus SA-1199B (Fig. 4H to N). The addition of glucose re-energizes the cells and promotes active efflux, and thereby reduced the efflux pump inhibitory potential of compounds (14) (Fig. S2). To further confirm the specificity of compounds as NorA efflux pump inhibitors, the EtBr efflux from S. aureus K1758 (*norA* deletion) was assessed. It was observed that none of the compounds significantly slowed down the EtBr extrusion both in the presence or absence of glucose. For the treated sets, no significant difference from the control group was observed (Fig. S3). Based on both the assays, the potential of the compounds to promote accumulation and inhibit the efflux of EtBr is in the order of pyrvinium >raloxifene >ezetimibe >propafenone ∼nefazodone ∼chlorprothixene.

**FIG 5:**
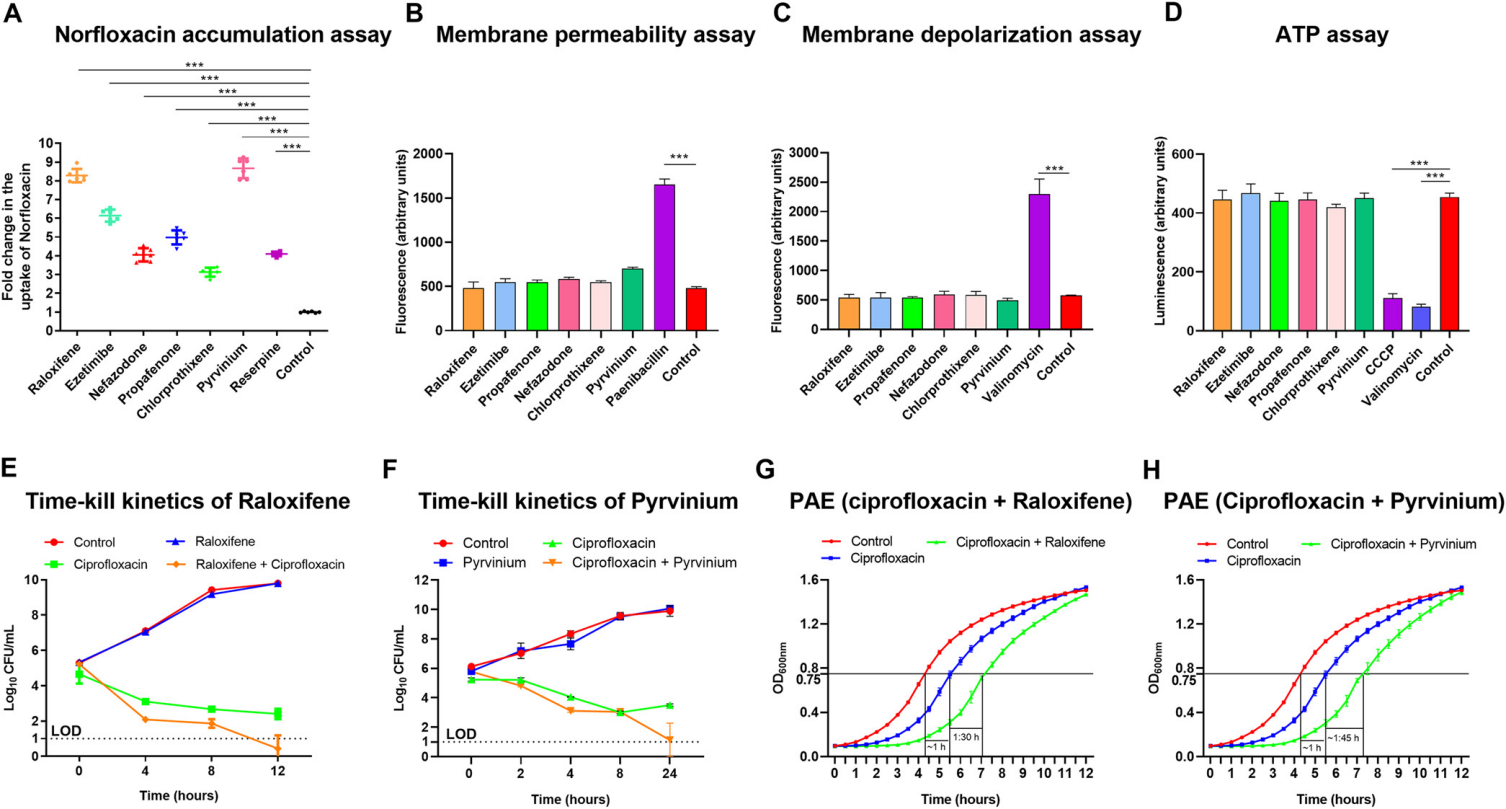
(A) Uptake of norfloxacin in S. aureus SA-1199B, when the cells were treated with raloxifene, ezetimibe, propafenone, nefazodone, chlorprothixene, pyrvinium, and reserpine at the sub-inhibitory concentrations (1/4 × MIC). The results presented correspond to the average of two independent experiments with three repeats ± SD. (B) Bacterial membrane permeability is measured with fluorescent PI dye upon exposure to the EPIs at 1 × MIC. Increased fluorescence corresponds to cells with the permeabilized membranes. Paenibacillin (20 μM) was used as a positive control since it destabilizes the Gram-positive bacterial membrane. The drug-free control was included to monitor change in fluorescence. (C) The EPIs do not depolarize the bacterial membrane. Shown is the change in fluorescence of S. aureus SA-1199B cells using a DiSC_3_(5) assay. Data is presented as a change in fluorescence before and after adding the EPIs and valinomycin at 1 × MIC. (D) S. aureus SA-1199B was exposed to the EPIs at 1/4 × MIC for 4 h. The ATP levels were quantified using a luciferin-luciferase bioluminescence detection assay. CCCP and valinomycin were included for comparison. The results presented correspond to the mean of three independent assays ± SD. (E) Time-kill curves of S. aureus SA-1199B showing the synergistic effect to enhance the antibacterial effect of ciprofloxacin (8 μg/ml) in combination with raloxifene (50 μM), (F) ciprofloxacin (8 μg/mL) in combination with pyrvinium (0.39 μM). Each time point represents the mean Log_10_ CFU/mL ± SD of three readings. LOD = Limit of detection for CFU. (G) The post-antibiotic effect (PAE) induced by ciprofloxacin (1 × MIC) alone and in combination with raloxifene (1/4 × MIC), (H) ciprofloxacin (1 × MIC) alone, and combination with pyrvinium (1/4 × MIC), for 2 h in S. aureus SA-1199B. Time 0 h corresponds to the beginning of growth monitoring immediately after compound removal and cell resuspension in fresh CA-MHB. The value of post-antibiotic effect corresponds to the delay undergone by the treated culture with respect to the untreated control in reaching an OD value half of the final OD. The assays were repeated three times independently, and the results were presented as mean ± SD. Results were considered highly significant when *** *P < *0.001.

Additionally, we performed a norfloxacin accumulation assay on S. aureus SA-1199B treated with EPIs to obtain more realistic evidence of the NorA efflux pump inhibition. The fluorescence measurement monitors the uptake of norfloxacin when it accumulates inside the cell (15). The results portray a significant increase in the norfloxacin accumulation in the EPIs (raloxifene, ezetimibe, propafenone, nefazodone, chlorprothixene, and pyrvinium) treated cells compared to reserpine and untreated control (Fig. 5A). The compound’s potential to promote norfloxacin accumulation is in the order of pyrvinium >raloxifene >ezetimibe >propafenone >nefazodone >chlorprothixene, based on norfloxacin accumulation assay.

### The EPIs do not alter membrane permeability.

The effect of compounds on membrane permeability of S. aureus SA-1199B was assessed using propidium iodide (PI) to rule out the likelihood of synergy due to any off-target effects. PI is a fluorescent dye that binds to double-stranded nucleic acids and is usually membrane-impermeable (12). Any alteration in membrane permeability caused by the presence of EPIs causes the dye to permeate cells and display an increase in fluorescence (16). No significant increase in fluorescence in the presence of EPIs (raloxifene, ezetimibe, propafenone, nefazodone, chlorprothixene, and pyrvinium) was observed, which indicates no membrane permeabilization. However, a drastic increase in fluorescence was observed in the cells treated with paenibacillin (positive control) (17) compared to the untreated control (Fig. 5B).

### The EPIs cause no membrane depolarization.

To determine the effect of EPIs on the membrane depolarization, we used 3, 3′-Dipropylthiadicarbocyanine iodide (DiSC_3_(5)), for assay against S. aureus SA-1199B. DiSC_3_(5) is a voltage-sensitive dye, a fluorescent molecular probe. The fluorescence of DiSC_3_(5) decreases as the dye partitions to the surface of polarized cells; depolarization prevents partitioning and can release bound dye into the media (18). None of the EPIs (raloxifene, ezetimibe, propafenone, nefazodone, chlorprothixene, and pyrvinium) had any significant effect on bacterial membranes, as they allowed stabilization of the quenched dye, revealing little to no depolarization. In contrast, the cells treated with valinomycin (positive control) displayed a drastic increase in fluorescence compared to untreated control, indicating membrane depolarization (Fig. 5C).

### The EPIs do not cause bacterial ATP depletion.

Any transition from normal membrane functions can destabilize the respiratory chain and lead to reduced ATP levels (12, 19). Any alteration in bacterial ATP synthesis levels 4 h post-treatment with EPIs (six-hit compounds) alone was evaluated to exclude the possibility of ATP depletion as EPIs functional mechanism. Carbonyl cyanide 3-chlorophenylhydrazone (CCCP) and valinomycin, known for energy depletion, were included as positive controls (20, 21). In the presence of EPIs, no depletion in the bacterial ATP levels was observed. In contrast, significant disruption of ATP synthesis in the CCCP and valinomycin treated cells was observed compared to untreated control (Fig. 5D).

### Effect of EPIs on time-kill kinetics of ciprofloxacin.

The time-kill kinetics of S. aureus SA-1199B was examined for 24 h to determine the enhanced antibacterial effect of ciprofloxacin in the presence of EPIs. Ciprofloxacin alone at 1 × MIC (8 μg/ml) and combined with raloxifene or pyrvinium (1/4 × MIC, i.e., 50 μM, 0.39 μM respectively) was used in the assay. As expected, both raloxifene and pyrvinium alone showed no antibacterial effect. Bacterial culture treated with ciprofloxacin alone (8 μg/ml) displayed killing in the initial 8 h; however, re-growth was observed afterward. A notable increase in the antibacterial activity of ciprofloxacin was observed after 8 h in the presence of both EPIs when compared to ciprofloxacin alone. The combinations of both raloxifene (Fig. 5E) and pyrvinium (Fig. 5F) almost entirely killed the bacteria in 12 h and 24 h, respectively, and maintained a difference of ∼ 4 log_10_ CFU below the initial log_10_ CFU at 0 h. Henceforth, ciprofloxacin-raloxifene and ciprofloxacin-pyrvinium duos could be potential anti-staphylococcal therapy.

### The EPIs prolonged the post-antibiotic effect (PAE) of ciprofloxacin.

According to a previous study (22), an antimicrobial agent causes PAE when—immediately after its removal—it brings about a growth delay of at least 0.5 h on a bacterial culture. A log-phase culture of S. aureus SA-1199B was treated with 1 × MIC of the ciprofloxacin in the presence and absence of raloxifene (50 μM) and pyrvinium (0.39 μM) for 2 h. The ciprofloxacin alone displayed a PAE of 1 h. In comparison, the ciprofloxacin–raloxifene (Fig. 5G) and ciprofloxacin–pyrvinium (Fig. 5H) duos increased the PAE of ciprofloxacin by 1.30 h and 1.45 h, respectively. Rationally, to ensure the success of the treatment, an EPI would not always need to be present, as long as the target bacteria retain sensitivity to the antibiotic (12). The prolonged PAE of the combination could aid in determining the optimum dosing frequency of the antibiotic by ensuring the widely-spaced dosing intervals (12).

### The EPIs reduced the resistant mutant development in response to ciprofloxacin.

Based on the above experiments, raloxifene and pyrvinium were found to be the most active EPIs, which prompted us to study the effect of the compounds on the resistant mutant development in response to the ciprofloxacin. We observed that raloxifene (50 μM) and pyrvinium (0.39 μM) decreased the mutation prevention concentration (MPC) of ciprofloxacin by 8- and 16-fold, respectively (Table 3). These results suggest the clinical utility of these combinations to limit the selection of spontaneous resistant mutants.

**TABLE 3:** Mutation frequency of ciprofloxacin alone, and in combination with raloxifene or pyrvinium against S. aureus ATCC 29213^*a*^

Compounds	Conc. used (μM)	Mutation frequency with ciprofloxacin					
		0.25 × mIC	0.5 × mIC	1 × mIC	2 × mIC	4 × mIC	8 × mIC
-	-	ND	ND	UC	UC	1.4 × 10^−11^	< 10^−11^
Raloxifene	50	ND	2.8 × 10^−11^	< 10^−11^	< 10^−11^	< 10^−11^	< 10^−11^
Pyrvinium	0.39	2.98 × 10^−11^	< 10^−11^	< 10^−11^	< 10^−11^	< 10^−11^	< 10^−11^

a ND, not determined; UC, uncountable colonies.

### The remarkable potency of EPI-ciprofloxacin combination on biofilm disruption.

**FIG 6:**
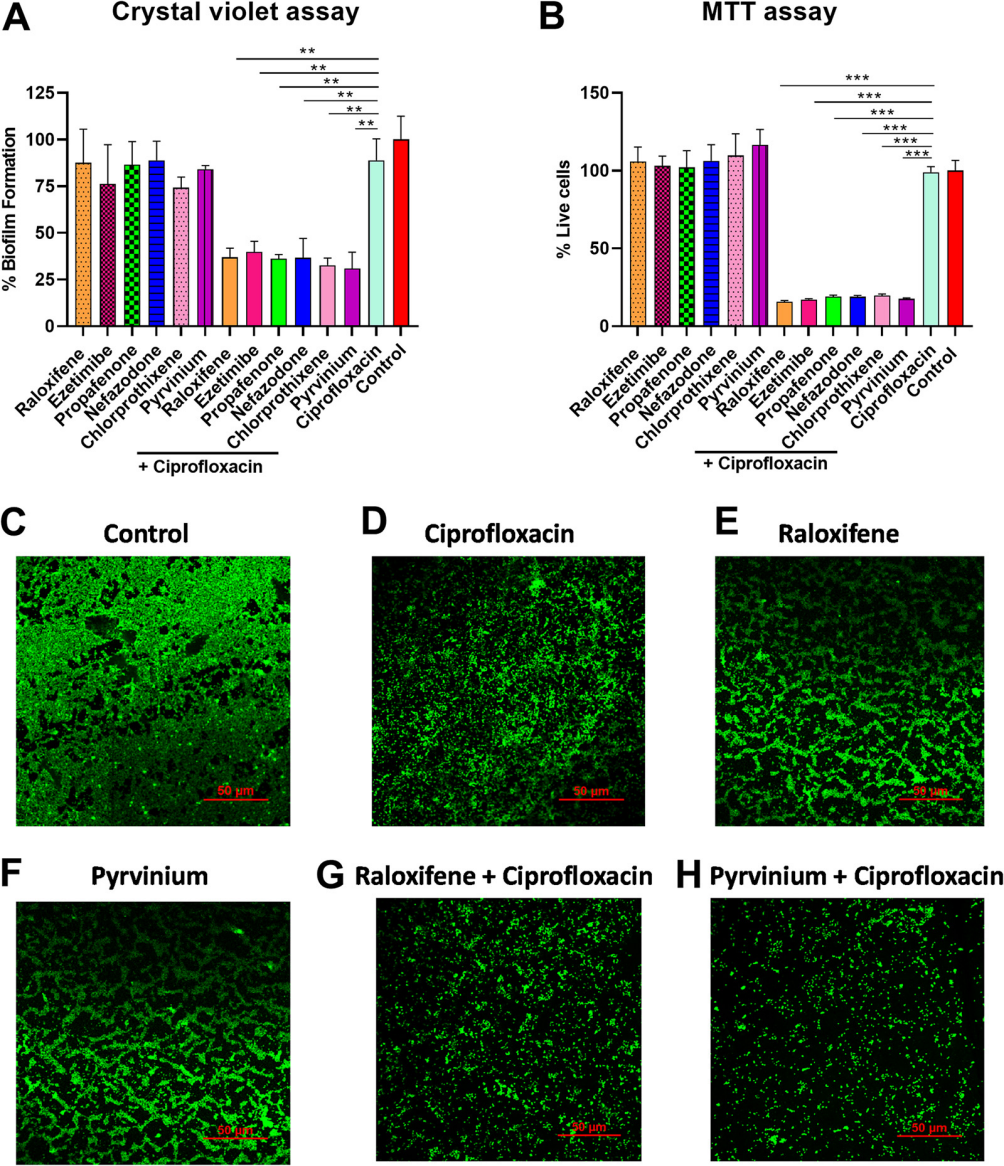
(A) Influence on the biofilm eradication ability of ciprofloxacin (8 μg/mL), EPIs (1/4 × MIC) alone, or in combination with the EPIs (1/4 × MIC); the crystal violet staining assessed the biomass of S. aureus SA-1199B after exposure to ciprofloxacin and ciprofloxacin-EPIs combination for 24 h. The percentage values represent the amount of biofilm formation with respect to drug-free control. The experiments were carried out in three biological repeats, and results correspond to average ± SD. Results were considered significant when *P < 0.05 and highly significant when ***P < 0.001. (B) Quantification of the effects of the EPIs at sub-inhibitory concentrations (1/4 × MIC) in combination with ciprofloxacin at 1 × MIC on mature biofilm; the viable bacterial cells were determined using MTT assay after exposure for 24 h. The percentage values represent the number of live cells with respect to drug-free control. The experiments were carried out in three biological repeats, and results correspond to average ± SD. Results were considered significant when **P < 0.01 and highly significant when ***P < 0.001. (C–H) Effect of ciprofloxacin (8 μg/mL) alone or in combination with raloxifene (50 μM) or pyrvinium (0.39 μM) on biofilm eradication assessed by confocal laser scanning microscopy (40×); static biofilms after exposure for 24 h were stained with SYTO9. S. aureus SA-1199B within biofilm on glass carriers display green fluorescence. The images are representatives of two independent experiments.

The strong potentiation in ciprofloxacin’s activity displayed by the EPIs prompted us to determine the effect of the EPI–ciprofloxacin duo on the preformed biofilms of S. aureus SA-1199B. In the crystal violet (CV) assay, treatment with EPIs (raloxifene, ezetimibe, propafenone, nefazodone, chlorprothixene, and pyrvinium) alone at sub-inhibitory concentrations (1/4 × MIC) exhibit negligible impact on the biofilm biomass. Moreover, ciprofloxacin (8 μg/ml) alone reduced the biofilm biomass by 11% only, while its combination with EPIs resulted in a significant reduction in biofilm biomass. The combination of EPIs with ciprofloxacin reduced the biofilm biomass by 63%, 60%, 63%, 63%, 67%, and 69%, respectively compared to the control (without treatment) (Fig. 6A).

The results of the CV assay made us anticipate the effect of the combinations on the cell viability in the mature biofilm. To our anticipation, we performed 3-(4, 5-dimethylthiazol-2-yl)-2, 5-diphenyl tetrazolium bromide (MTT) colorimetric assay. Unlike CV, the MTT dye does not stain biological molecules like DNA, proteins, polysaccharides, and others within the bacterial biofilm. The MTT assay measures the metabolic activity of an individual bacterial cell in the biofilm, and only live cells are stained. The bacterial viability is determined by the absorbance at 570 nm, which correlates with the formazan concentrations (23). The EPIs alone at sub-inhibitory concentrations (1/4 × MIC) did not significantly impact the viable cell population of the preformed biofilm (Fig. 6B). The treatment with ciprofloxacin alone (8 μg/ml) displayed negligible effect by decreasing the viability of biofilm by 1.05%, while the ciprofloxacin-EPI combination resulted in a significant decrease in cell viability. The combination of ciprofloxacin with the hit EPIs, i.e., raloxifene, ezetimibe, propafenone, nefazodone, chlorprothixene, and pyrvinium, decreased the live cell population in preformed biofilm by around 80%–84%, compared to the untreated control (Fig. 6B).

Further, we visualized the effect of raloxifene and pyrvinium (best EPIs) in combination with ciprofloxacin on biofilm structure by confocal microscopy supplemented with SYTO9 stain. SYTO9 is a cell-permanent nucleic acid stain and shows a significant fluorescence enhancement when it binds nucleic acids. The regions with SYTO9 staining show green fluorescence and represent live bacterial cells of biofilm. The biofilm of untreated control showed a sturdy structure, and for ciprofloxacin, raloxifene, and pyrvinium alone, a minimal disruption was observed. In contrast, the combination of raloxifene (50 μM) and pyrvinium (0.39 μM) with ciprofloxacin (8 μg/ml) caused significant disintegration of the biofilm (Fig. 6C to H).

### The efficacy of the combination in a murine thigh infection model.

**FIG 7:**
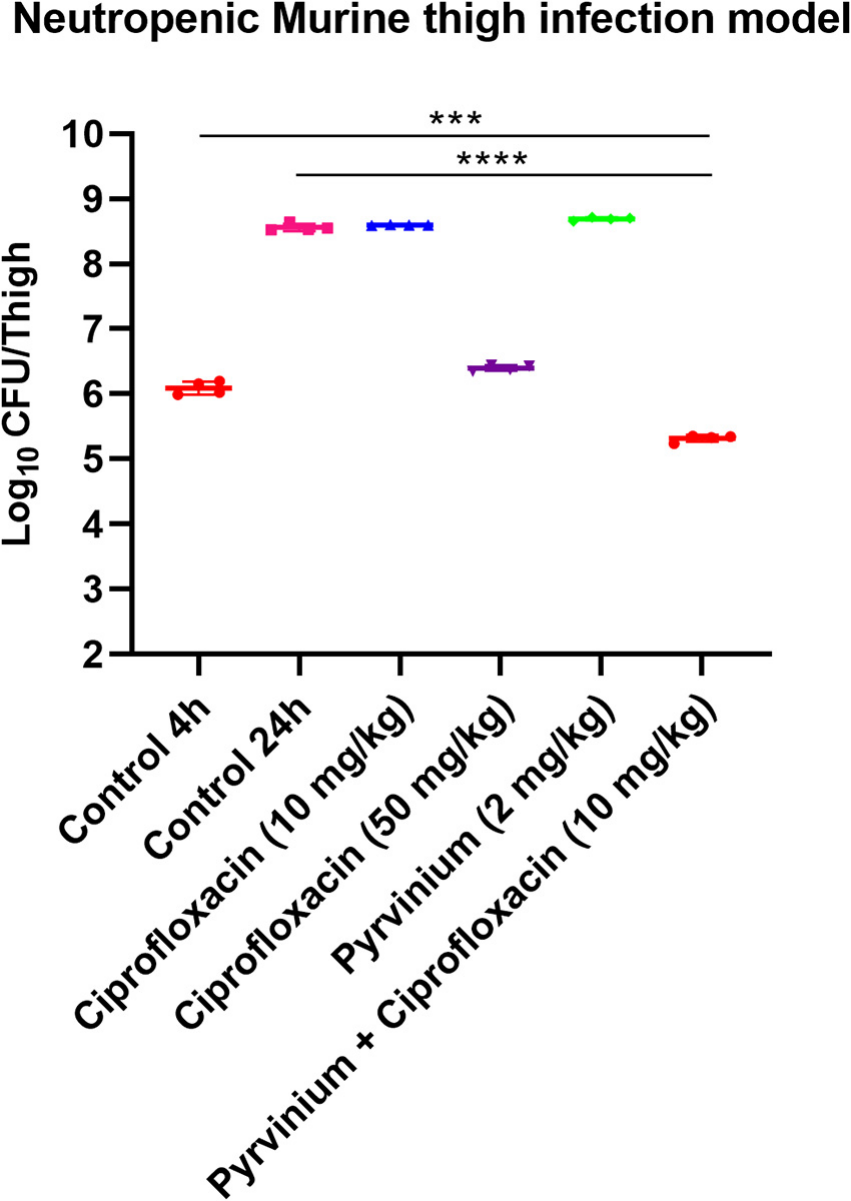
Efficacy of pyrvinium/ciprofloxacin combination in a neutropenic murine (BALB/c female, age: 6–7 weeks, weight: 20 g) thigh infection model; single-dose (subcutaneous; 4 h after infection, 4 mice per group) treatment with ciprofloxacin, pyrvinium alone, and in combination against S. aureus SA-1199B; for drug-treated animals, CFU/thigh was determined at 24 h after infection. For controls, CFU/thigh was estimated at 4 h and 24 h after infection. The CFU/thigh is plotted as individual points, and error bars represent the SD within an experimental group.

The potent *in vitro* synergistic activity of pyrvinium suggested that this molecule, combined with ciprofloxacin, could be used to treat severe bacterial infections in clinical settings. We determined the *in vivo* efficacy of ciprofloxacin combined with pyrvinium in a neutropenic murine thigh infection model. The treatment of neither ciprofloxacin (50 mg/kg), ciprofloxacin (10 mg/kg), nor pyrvinium (2 mg/kg) alone exhibit a significant reduction in bacterial counts. In contrast, the ciprofloxacin and pyrvinium combination proved to be very efficient, resulting in ∼ 0.79 log_10_ and 3.2 log_10_ reductions in CFU compared to the untreated control at 4 h and 24 h, respectively (Fig. 7).

## DISCUSSION

Efflux pumps are proteinaceous membrane-integrated transporters in charge of expelling various substances that are otherwise harmful to bacterial survival. Multidrug-resistant (MDR) efflux pumps play a prominent role in drug resistance, pathogenicity, and, as recently described, also in biofilm formation (24). NorA is an MDR efflux pump of the MFS, and expels various hydrophilic fluoroquinolones and dyes in S. aureus. NorA is well established as a model system for studying efflux inhibition in S. aureus. Considering the higher percentage of NorA overproduction in clinical S. aureus strains, this study aimed to investigate potential new EPIs. By limiting the extrusion of antibiotics, EPIs raise their intracellular accumulation at the same dose of the antibiotic. As a consequence, antibiotic-resistant bacteria may become susceptible to that particular antibiotic. Many EPIs have been discovered in past years, but none is clinically approved and achieves optimum plasma concentrations. Thus, we screened FDA-approved drugs for potential NorA EPIs. Our approach was previously validated in a recent study by screening the FDA-approved drug library and identifying nilotinib as the first clinically approved drug that acts as NorA EPI, achieves plasma levels, and displays anti-biofilm activity. However, the *in vivo* efficacy studies have not been demonstrated hitherto to the best of our knowledge (25).

The library of FDA-approved drugs was docked to a NorA homology model to identify initial hits. Reserpine, a well-known NorA inhibitor, was predicted to possess binding energy of –4.661 kcal/mol. Since the molecular modeling was performed using homology models, the results will depend on the accuracy of the models used in the study. The templates we used for homology models show high sequence similarity with transporters belonging to MFS. The hits obtained docks at the conserved domain (26), which is the potential substrate, i.e., ciprofloxacin binding site hence suggesting a competitive mode of inhibition. The four FDA-approved drugs, i.e., raloxifene, ezetimibe, propafenone, and nefazodone, having higher binding energies than reserpine, showed equal or greater synergistic activity with ciprofloxacin. Compounds with relatively weak binding energies (chlorprothixene and pyrvinium) also showed strong synergism. Thus, our results suggest a moderate correlation between binding energy and EPI activity.

An ideal EPI should lack intrinsic antibacterial activity as bacteria may develop resistance against them (27). The absence of antimicrobial activity at used concentrations makes the compounds in this study advantageous for use as putative EPIs. Compounds with an effect similar to or better than reserpine were tested for synergism. All of the six compounds increased the susceptibility of *norA* overexpressing S. aureus to ciprofloxacin and norfloxacin by many folds in a concentration-dependent manner. It was mentioned earlier that a reduction in the MICs by a minimum of 4-fold with respect to their original values in the presence of a potentiator or an EPI was considered an indication of efflux inhibition (28). Additionally, there was no reduction in MICs of S. aureus K1758, which lacks the functional *norA* gene. It represents the specificity of these compounds as putative NorA EPIs. The compounds demonstrated synergism with ciprofloxacin and decreasing its MICs below or equal to the clinical resistance breakpoint (1 mg/L) in *norA* over-expressing as well as some clinical strains of S. aureus. Moreover, raloxifene, ezetimibe, and pyrvinium demonstrated synergism with norfloxacin. They decreased its MIC to the clinical resistance breakpoint (4 mg/L) in *norA* over-expressing strain, where propafenone, nefazodone, and chlorprothixene demonstrated synergism but could not reduce its MIC to the clinical resistance breakpoint. The bactericidal activity in an agar-plate assay seeded with S. aureus SA-1199B indicates the ability of compounds to potentially reverse NorA efflux pump mediated resistance, facilitating the treatment of ciprofloxacin-resistant S. aureus.

EtBr accumulation and efflux inhibition assays indicate the efflux pumps’ contribution to resistance (26). The EtBr accumulation and efflux inhibition assay results portray inhibited efflux and increased uptake of EtBr in the presence of all six EPIs. Next, the uptake of norfloxacin was determined in the presence of compounds to test if NorA inhibition could increase its accumulation within the bacterial cell. The natural fluorescence of norfloxacin is directly correlated with its concentration inside the bacterial cell. The results suggested that compounds increased the concentration of norfloxacin inside the S. aureus SA-1199B. These results provided evidence to support the specificity of putative NorA EPIs discovered herein. However, the effect of compounds upon other MDR efflux pumps extruding EtBr and fluoroquinolones cannot be ruled out.

None of the compounds except chlorprothixene has earlier been reported as EPIs against S. aureus. Chlorprothixene has been reported to reduce or reverse the resistance of Gram-positive and Gram-negative bacterial strains to various antibiotics to which these strains were initially resistant (29).

Many EPIs discovered earlier have several toxicity issues due to their off-target effects. Disruption and depolarization of the bacterial cell membrane may cause efflux inhibition through a nonspecific effect leading to the common identification of false-positive EPIs (12, 30). Also, the capacity of a bacterial cell to synthesize ATP is closely related to the loss of membrane integrity (31). In our study, none of the EPIs, at least at working concentrations, either disturb the membrane function or deplete the bacteria’s total ATP pool. Conclusively, the synergism displayed by the drugs indicates the specificity and true EPI-likeness of the drugs and is not an outcome of any off-target effects.

In time-kill kinetics, *synergy* is defined as a reduction of 2 log_10_ CFU/mL in combination compared to its most active counterpart (antibiotic here), and must be ≥2 log_10_ CFU/mL below the starting inoculum (12, 32). We observed 1.9 log_10_ and 2.3 log_10_ reductions in CFU for raloxifene and pyrvinium combinations, respectively, compared to ciprofloxacin alone. The combination of pyrvinium and ciprofloxacin exhibited synergy. Therefore, we determined the PAE of ciprofloxacin in combination with raloxifene and pyrvinium. Moreover, raloxifene and pyrvinium boosted the PAE of ciprofloxacin by ≥1.30 h (∼150% increment). A similar observation about the enhanced PAE of ciprofloxacin in the presence of capsaicin has been reported previously (26). These results may have significant clinical implications if an EPI causing PAE enhancement is intended to be co-administered with an antibiotic. The basis of the current condition of antimicrobial resistance is the rapid development of bacterial resistance against any antibiotic. Perhaps, bacterial EPIs do not target the essential bacterial processes; they can avoid the resistance development against them. Notably, it has been shown that the non-antibacterial adjuvants that inhibit the resistance mechanism of that antibiotic can lead to diminished resistance development (33). Likewise, we observed a notable decrease in the frequency of spontaneous resistant mutant development in response to ciprofloxacin when combined with raloxifene and pyrvinium. Studies have reported that several similar EPIs, such as capsaicin and boeravinone B, significantly reduced the emergence of ciprofloxacin-resistant mutants of S. aureus (26, 34).

Bacterial biofilms are associated with a large number of difficult-to-treat infections. Active efflux pumps have been reported to be upregulated and increase resistance in mature biofilms (35, 36). The inhibition of efflux pumps has also been previously reported to reduce biofilms (37). Moreover, EPIs are previously described to significantly reduce preformed biofilms (24). It makes efflux pumps potentially valuable targets for anti-biofilm measures. Thus, we investigated the effect of EPIs and their combination with ciprofloxacin to eradicate biofilm. The exact contribution of the NorA efflux pump in biofilm formation is not yet fully understood. In our study, EPIs and ciprofloxacin alone did not cause the eradication of biofilms. So, we hypothesized that the effect of EPIs on biofilms might not be due to a direct disruption but rather due to the inhibition of NorA, leading to an accumulation of ciprofloxacin. Some earlier studies reported the potential of raloxifene and pyrvinium as EPI or anti-biofilm compounds in eukaryotes or fungi (38, 39).

Out of all six EPIs discovered in our study, raloxifene and pyrvinium are the two best hits based on the *in vitro* studies. Raloxifene is prescribed for the treatment and prevention of osteoporosis in postmenopausal women, and reduced risk of invasive breast cancer in postmenopausal women with osteoporosis; it was previously reported to reduce biofilm formation. The possible side effects indicated during raloxifene treatment are joint pain, leg cramps, hot flashes, feet, ankles, swelling of the hands and lower legs, flu-like syndrome, difficulty falling asleep, sweating, or staying asleep (40). Pyrvinium (a quinoline-derived cyanine dye) is a relatively non-toxic anthelminthic drug, FDA-approved, used to treat pinworms in humans and animals. The possible side effects are rare, including skin rash, diarrhea, increased skin sensitivity to sunlight, nausea, vomiting, and stomach cramps (41).

The next aim of the study was to investigate if the EPI activities observed *in vitro* would also display activity in a murine model. None of the approved drugs discovered so far have been known to reach sufficient plasma levels as well as demonstrated *in vivo* efficacy as NorA EPI. The concentration of pyrvinium in the plasma ranged from approximately 150 nM to 500 nM using a once-daily dosing regimen (42)—well within its *in vitro* efficacy range. This information indicated it was feasible to study the *in vivo* efficacy of pyrvinium as an anti-efflux drug. We observed remarkable efficacy of the pyrvinium–ciprofloxacin combination in reducing S. aureus burden inside the thigh muscles of mice compared to untreated control and ciprofloxacin alone.

In conclusion, the hit compounds identified in this study have the potential as a therapeutic adjuvant to rejuvenate the activity of fluoroquinolones toward S. aureus. The EPIs appear to act in a specific manner and possess no apparent undesirable side effects of previously described EPIs. Interestingly, pyrvinium was observed to be effective on preformed biofilm and has an excellent *in vivo* efficacy at clinically achievable concentrations. We suggest the pyrvinium–ciprofloxacin combination suitable for the mitigation of MDR S. aureus infections. However, further *in vivo* studies are required to determine the appropriateness of this adjunctive therapy.

## MATERIALS AND METHODS

### Chemicals, bacterial strains, and growth conditions.

All the chemicals, antibiotics, and dyes used in the study were purchased from Sigma-Aldrich Chemical Co. (St. Louis, MO, United States) unless mentioned otherwise. The strains S. aureus SA-1199B (*norA* over-expressed) (43), S. aureus SA-1199 (43), and SA-K1758 (*norA* deletion mutant of NCTC 8325-4) (44) were used in the study. The S. aureus clinical strains MRSA 1, MRSA 2, MRSA 3, MRSA 4, GMCH 831, and GMCH 839 were collected from Government Medical College and Hospital, Chandigarh, India. The tetracycline-resistant clinical isolate S. aureus XU212 having a *tet(K)* efflux pump expression (45), was also used in the study. The S. aureus ATCC 29213 was purchased from Himedia, India. All the strains were grown under standard culture conditions at 37°C in BBL Cation-adjusted Mueller-Hinton Broth (CA-MHB; B.D., U.S.) unless specified otherwise. For CFU counting Mueller-Hinton Agar (MHA) (Himedia, India) was used.

### Homology modeling of NorA and docking studies.

The homology modeling of the NorA protein and the receptor preparation for docking were performed as described previously (46). The three-dimensional structure of NorA protein was predicted using PDB IDs 3WDO, 4ZOW, 4ZPO, and 6EXS as templates by I-TASSER online server (47). The FDA-approved drug library was downloaded from the DrugBank database (www.drugbank.ca) and prepared for docking study using the LigPrep module (48) in the Schrodinger software suite. The potential drug binding sites in the modeled structure were identified using the SiteMap (49). The SiteMap analysis identified one potential druggable site with a score of >1 located at the binding site of the predicted NorA structure. This site was used for grid generation for docking by keeping box dimension 12 Å. The molecular docking studies and binding affinity calculations were performed using the Glide module and MM-GBSA modules (50), respectively, in the Schrodinger software suite 2019. The molecular graphics figures for this study were prepared using PyMOL (51).

### MIC determination.

MICs were determined according to the Clinical & Laboratory Standards Institute (CLSI) guidelines using Broth-microdilution assay in CA-MHB (52). For the assay, from 10 mM master stocks, working solutions of 1 mM were prepared in DMSO. From 1 mM working solutions, different concentrations of drugs were tested to determine the MIC of the drugs. A control equivalent to the final concentration of DMSO/well was also tested to avoid any inhibitory activity of drugs due to the DMSO effect. The 2-fold serial dilutions of the compounds were loaded to wells of 96-well polystyrene tissue culture plates (Corning, U.S.) and further inoculated with 5 × 10^5^ CFU/ml of the bacterial culture. Then, the plates were incubated at 37°C for 18 h. The wells containing a minimum concentration of the compound with no visual turbidity were considered the compound’s MIC.

### Determination of growth kinetics.

The freshly grown S. aureus SA-1199B culture was prepared into a bacterial suspension of a final density of 5 × 10^5^ CFU/ml in CA-MHB. The bacterial suspension was cocultured with sub-inhibitory concentrations of raloxifene (50 μM), ezetimibe (50 μM), propafenone (50 μM), nefazodone (50 μM), chlorprothixene (50 μM), and pyrvinium (0.39 μM). The culture without treatment was included as a control for comparison. The absorbance (OD_600nm_) based measurements of cell growth densities were performed at an interval of 1 h from 0 h to 21 h.

### Checkerboard synergy assay.

The synergistic activity of compounds with antibiotics was determined using broth microdilution assay. Briefly, the 2-fold serial dilutions of antibiotics were loaded into the wells of 96-well polystyrene tissue culture plates down the assay plate, and varying concentrations of compounds were loaded across the assay plates. The wells containing antibiotic + compound cocktail were finally inoculated with 100 μL of 5 × 10^5^ CFU/ml bacterial culture and incubated for 18 h at 37°C. Fractional Inhibitory Concentration Index (FICI) values were determined according to the following formula:

FICI = FICI_A_ + FICI_B_

FICI_A_ = MIC of compound A in combination/MIC of compound A alone

FICI_B_ = MIC of compound B in combination/MIC of compound B alone

The FICI value of ≤ 0.5 represents “Synergy,” whereas 0.5 < FICI ≤ 4.0 was the “indifference” effect, and the FICI > 4.0 represents an “antagonism” (12, 53).

### Agar plate assay.

For the assay, S. aureus SA-1199B was grown to OD_600nm_ of 0.3 and spread onto MHA plates at the final inoculum of 10^5^ CFU/mL using cotton swabs. The individual discs containing sub-inhibitory concentration (1/4 × MIC) of compounds, ciprofloxacin (0.5 μg/mL, 1 μg/mL, 8 μg/mL) alone and a combination of both were placed on the surface of agar spread-plates. The plates were incubated for 24 h at 37°C and assessed for growth inhibition zone. The zones of growth inhibition around the discs were measured with the HiAntibiotic Zone scale.

### Ethidium bromide accumulation assay.

The assay was performed as described previously (12, 26), with some modifications. Briefly, the S. aureus SA-1199B was grown to the log phase (OD_600nm_ ∼ 1.0) and centrifuged for 15 min at 16,060 × *g*. The pellet was washed twice and resuspended in uptake buffer (110 mM NaCl, 7 mM KCl, 50 mM NH_4_Cl, 0.4 mM Na_2_HPO_4_, 52 mM Tris base; pH 7.5). The culture was diluted to OD_600nm_ of 0.3, then loaded with EtBr (4 μg/mL) and treated with compounds at sub-inhibitory concentrations (1/4 × MIC, 1/8 × MIC, and 1/16 × MIC). Reserpine (32 μg/mL) was included as a positive control. The increase in fluorescence at an emission wavelength of 600 nm by exciting at 530 nm was recorded for 45 min (9 min time-interval) in a microplate reader (BioTek, USA). The concentration-dependent curves were plotted for each compound.

### EtBr efflux inhibition assay.

The EtBr efflux inhibition assay was performed by a method described earlier (12, 14). The S. aureus SA-1199B was grown at 37°C until the OD_600nm_ reached 1.0. The culture was pelleted for 10 min at 16,060 × *g*, washed twice with Phosphate buffered saline (PBS; pH 7.4), and diluted to OD_600_ of 0.4 in PBS. Further, the bacterial suspension was loaded with EtBr (4 μg/mL), treated with compounds at sub-inhibitory concentrations (1/4 × MIC), then incubated at 25° C for 60 min to allow maximum accumulation to occur. After maximum accumulation occurred, bacteria were pelleted for 15 min at 16,060 × *g* and then resuspended in PBS. Finally, the aliquots were loaded to 96-well black tissue culture plates (Corning, U.S.). The fluorescence was recorded for 30 min (6 min time interval) at excitation and emission wavelength of 530 nm and 600 nm, respectively, in a microplate reader (BioTek, USA). The change in fluorescence was also determined in the presence and absence of 0.4% glucose.

### Norfloxacin accumulation assay.

The S. aureus SA-1199B was grown to an OD_600nm_ of 0.6 and treated with norfloxacin (64 μg/ml) alone or in combination with compounds at sub-inhibitory concentrations (1/4 × MIC). Reserpine (32 μg/ml) was included as a positive control (10). The treated bacterial culture samples were incubated on ice for 15 min, centrifuged, washed with sodium phosphate buffer (pH 7.4), and then resuspended in 1 ml of glycine-HCl buffer (pH 3.0). Further, the culture samples were incubated at room temperature for 2 h, followed by centrifugation. The fluorescence of 1:10 dilution of the supernatant was measured at excitation and emission wavelengths of 281 nm and 440 nm, respectively, in a microplate reader (BioTek, USA) (54).

### Membrane permeabilization assay.

The assay was performed as described earlier (12), with some modifications. The bacterial culture of S. aureus SA-1199B was grown to the mid-log phase (OD_600nm_ ∼ 0.5) in CA-MHB. It was centrifuged at 10,000 × *g* for 10 min, washed thrice in 0.85% saline, and final density was prepared to OD_600nm_ ∼ 0.4. The bacterial suspension was incubated with compounds (1 × MIC) and paenibacillin (20 μM) for 1 h at 37°C. Paenibacillin was included as a positive control as it is known to destabilize the Gram-positive bacterial membrane (17). To remove the effect of compounds, the treated culture was centrifuged at 10,000 × *g* and resuspended in saline. Further, the PI at a final concentration of 30 μM was added to a 96-well polystyrene black flat-bottom plate containing cells treated with compounds. The fluorescence was recorded for 24 min (3 min time interval) in a microplate reader (BioTek, USA) at the excitation and emission wavelength 490 nm and 635 nm, respectively.

### Membrane depolarization assay.

We performed a DiSC_3_(5) assay to determine the effect of compounds on membrane depolarization, as described earlier (12, 55). DiSC_3_(5) is a membrane-potential sensitive probe. Briefly, the S. aureus SA-1199B was grown to log phase (OD_600nm_ ∼ 0.5), centrifuged. Further, the cells were washed twice in 5 mM 4-(2-hydroxyethyl)-1-piperazineethanesulfonic acid (HEPES) buffer (containing 100 mM KCl, pH 7.2) to equilibrate the cytoplasmic and external K^+^ ion concentrations. The cells were prepared to OD_600nm_ ∼ 0.4 and incubated with DiSC_3_(5) (0.4 μM) for 60 min at 37°C. Further, the bacterial suspensions were incubated with compounds (1 × MIC) and valinomycin (16 μg/ml) in a 96-well black flat-bottom plate for 30 min. The variation in fluorescence was recorded at an excitation and emission wavelength of 622 nm and 670 nm, respectively.

### Determination of intracellular ATP levels.

The effect of compounds on the intracellular ATP levels was determined using an ATP determination kit (Invitrogen, Life Technologies, U.S.). As described elsewhere (46), the bacterial culture S. aureus SA-1199B was grown up to the mid-log phase (OD_600nm_ ∼ 0.5), and the final OD_600nm_ was adjusted to 0.3. The bacterial culture was treated with sub-inhibitory concentrations of compounds (1/4 × MIC) for 4 h at 37°C. After treatment, the cells were lysed first in an ultrasonic water bath and subsequently inactivated by heat-cold shock. The persistent ATP from cell lysate was measured in a 96-well black flat-bottom plate and plotted as relative luminescence units. The culture suspensions treated with CCCP (16 μg/mL) and valinomycin (4 μg/mL) were included as positive controls.

### Time-kill kinetics.

The bacterial culture of S. aureus SA-1199B corresponding to a final density of 5 × 10^5^ CFU/mL was treated with ciprofloxacin (8 μg/mL) alone and in combination with raloxifene (50 μM) or pyrvinium (0.39 μM). The culture without treatment was included as a control. All the treatment sets were maintained at an equal volume of 1 ml for each time point and incubated at 37°C with shaking, and 100 μL of the sample was withdrawn from each set at different time points and spread onto MHA plates. The spread plates were incubated for 24 h at 37° C, colonies were counted, and CFU/mL was assessed. Limit of Detection (LOD) is the lowest amount of analyte (microorganisms here) in a sample that can be detected with probability, although perhaps not quantified as an exact value (56).

### Determination of the PAE by turbidimetry.

The bacterial culture of S. aureus SA-1199B was grown to OD_600nm_ ≈ 0.3, incubated with ciprofloxacin (8 μg/mL) alone, and combined with raloxifene (50 μM) or pyrvinium (0.39 μM) for 2 h at 37°C (12). The 1 mL treated culture suspensions were diluted 50 times to eliminate the drug carryover, and an aliquot of 250 μL was loaded to a 96-well flat-bottom microtiter plate (Cole-Parmer, U.S.) in triplicates. The value of PAE corresponds to the treatment culture’s delay in reaching an OD value of half of the final OD compared to the untreated control (12). The untreated control was included for the calculation of PAE. PAE was calculated according to the formula PAE = T_50_ – C_50_, where T_50_ and C_50_ are the time in hours required for the drug-treated and untreated cultures, respectively, to reach a value of OD_600nm_ corresponding to 50% of the final absorbance reached by an untreated control (57). The control set’s cellular concentration was adjusted equal to treated suspension before growth resumption to minimize the difference in the inoculum.

### Mutation prevention concentration determination.

The S. aureus ATCC 29213 was grown to log phase (OD_600nm_ ∼ 1.0), and 100 μL from total culture volume of 5 mL (∼10^8^ CFU) was spread on MHA plates containing different concentrations of antibiotic alone and antibiotic in combination with the sub-inhibitory concentration of EPIs. The plates were incubated at 37°C for 48 h and then examined for the number of colonies. The concentration at which no colony appeared was considered as the MPC of the antibiotic (58). The mutation frequency was calculated as the formula Mutation frequency = number of survivors/CFU plated.

### Biofilm eradication assay.

According to a method described earlier (12, 59), the biofilm eradication assay was performed with some modifications. The S. aureus SA-1199B culture was grown overnight, diluted 1:200 times in fresh Tryptic soy broth (TSB) supplemented with 1% glucose, and loaded into wells of a 96-well flat-bottom plate (Falcon 96-well Polystyrene Microplates; tissue culture treated). The plate was incubated at 37˚ C for 48 h under static conditions. Further, the wells were washed twice using PBS to remove the non-adherent planktonic cells and treated with ciprofloxacin (8 μg/mL) and compounds (1/4 × MIC) alone and in combination. Post-treatment, the plate was incubated for 24 h at 37˚ C; further wells were washed with PBS and fixed with 99% methanol for 15 min. The plate was dried for a few min in laminar airflow, and the adherent bacteria in the biofilm were stained with filtered 0.1% crystal violet (CV) for 5 min at room temperature. The surplus stain was removed by washing with water until the negative control wells (without biofilms) appeared colorless. Then, the cells carrying the stains were incubated with 33% acetic acid to solubilize the stain, and the absorption of the released stain was recorded at 590 nm. The results are plotted as the percentage biofilm formation with respect to the untreated control group.

### Determination of viable cell population in biofilm.

For quantifying live cells in the biofilm, MTT assay was performed (12, 23) according to the method described earlier. The mature biofilm was grown as described in the CV assay. After cell fixation, MTT (1 mg/mL) was added, and plates were incubated for 3–4 h at 37°C. The cells were washed once to release surplus dye and solubilized using a solution (40% (vol/vol) dimethylformamide in 2% (vol/vol) glacial acetic acid added with 16% sodium dodecyl sulfate). The absorbance of the released stain was recorded at 570 nm. The results indicate the percentage of viable adherent bacteria present in the biofilm with respect to the untreated control group.

### Confocal microscopy for analysis of biofilm eradication.

The bacterial culture was prepared similar to the CV assay (12, 60). The coverslips with poly-L-Lysine coating were used to ensure biofilm attachment. The coverslips were placed in 12-well polystyrene tissue culture plates (Corning, U.S.) with coated side upward, and the wells were dispensed with 1 mL of the bacterial suspension. The plates were incubated under static conditions for 48 h at 37°C. After 48 h, wells were washed twice with 1× PBS, treated with ciprofloxacin (8 μg/mL) alone and in combination with raloxifene (50 μM) or pyrvinium (0.39 μM) in fresh TSB, and the plates were again incubated for 24 h. The wells without treatment served as a control. Post-treatment, the media were discarded, and the wells were washed thrice with 0.85% saline and then stained with SYTO 9 (Invitrogen, Life Technologies, U.S.) dye for 35–40 min. After staining, the wells were washed once to remove the surplus dye and dried for a few minutes in laminar airflow. The biofilm-coated coverslips were mounted inverted on the slides using mounting oil. The stained biofilm slides were analyzed using inverse confocal laser scanning microscopy (CLSM) (Nikon AI(R)) at 488 nm excitation. The experiment was performed in duplicates, and images were visualized and processed using NIS-Elements Viewer 4.50 software.

### Ethics statement.

This study was carried out according to the recommendations of the Committee for the Purpose of Control and Supervision of Experiments on Animals (CPCSEA). The protocol was approved by the Institutional Animal Ethics Committee (IAEC/20/04) of the CSIR-Institute of Microbial Technology, Chandigarh, India. Neither randomization nor blinding was considered necessary for the animal infection models.

### *In vivo* murine thigh infection model.

Female BALB/c mice (age: 6–7 weeks, weight: 20 g) (4 in each group) were made neutropenic by cyclophosphamide: first dose (150 mg/kg) 4 days and second dose (100 mg/kg) 1 day before infection. A bacterial culture of 10^7^ CFU/mL of S. aureus SA-1199B was resuspended in sterile PBS, and a 50 μL volume was administered via the intramuscular route in the right thigh. A combination of ketamine (90 mg/kg) and xylazine (10 mg/kg) was injected via the intraperitoneal route prior to cervical dislocation. One group was euthanized by cervical dislocation at 4 h and another at 24 h postinfection. The right thighs were removed aseptically, homogenized into PBS, serially diluted, and plated for CFU counts on MHA. The remaining group of mice was treated with either pyrvinium (2 mg/kg) or ciprofloxacin (10 mg/kg and 50 mg/kg) alone or, in combination, injected subcutaneously (four mice per group). At 20 h post-treatment, the mice were euthanized, and CFU per thigh were calculated.

### Statistical analysis.

All experiments were performed in biological replicates, and similar results were obtained on all occasions. The data are represented as mean ± standard deviation (SD). Multiple mean *t*-tests (two-tailed) were used to determine the differences among groups by GraphPad Prism 8.0.2 software package. A *P* value < 0.05 was considered statistically significant and highly significant when *p* < 0.01 and *p* < 0.001.
