# Supplementary material for: Repurposing Approved Drugs as Fluoroquinolone Potentiators to Overcome Efflux Pump Resistance in Staphylococcus aureus
Source: Microbiol Spectr. 2021 Dec 15;9(3):e00951-21. doi: 10.1128/Spectrum.00951-21 (PMC8672906; doi:10.1128/Spectrum.00951-21)
Supplement: SUPPLEMENTAL FILE 1 — Supplemental material. Download SPECTRUM00951-21_Supp_1_seq6.pdf, PDF file, 0.7 MB [file spectrum00951-21_supp_1_seq6.pdf]

SUPPLEMENTARY INFORMATION

Repurposing Approved Drugs as Fluoroquinolone Potentiators to Overcome Efflux Pump Resistance in *Staphylococcus aureus*

Nisha Mahey[1, 2], Rushikesh Tambat[1], Nishtha Chandal[1, 2], Dipesh Kumar Verma[3], Krishan Gopal Thakur[3], Hemraj Nandanwar[1, 2,*]

[1]Clinical Microbiology & Antimicrobial Research Laboratory, CSIR-Institute of Microbial Technology, Sector 39-A, Chandigarh-160036, India

[2]Academy of Scientific & Innovative Research (AcSIR), Ghaziabad, Uttar Pradesh-201002, India

[3]Structural Biology Laboratory, CSIR-Institute of Microbial Technology, Sector 39-A, Chandigarh-160036, India

[*]ADDRESS FOR CORRESPONDENCE

Dr. Hemraj Nandanwar, Senior Principal Scientist, CSIR-Institute of Microbial Technology, Sector 39-A, Chandigarh-160036, India

E-mail ID: hemraj@imtech.res.in

Telephone: +91-172-6665338   Fax: +91-172-2690585/2690632

RUNNING TITLE

Repurposing drugs for resistant *S. aureus*

.

SUPPLEMENTARY INFORMATION (LEGENDS)

Table S1 Binding energy (kcal/mol) of the lead compounds to the homology model of NorA efflux pump.

Table S2 MIC of ciprofloxacin, norfloxacin in combination with compounds for *S. aureus* SA1199 (Wild-type strain).

Table S3 MIC of tetracycline and moxifloxacin for *S. aureus* XU212 and *S. aureus* SA-1199B respectively in combination with different drugs.

Table S4 MIC of moxifloxacin for *S. aureus* clinical strains (MRSA) in combination with different drugs.

Fig. S1 Growth kinetics of *Staphylococcus aureus* SA-1199B in CA-MHB under sub-inhibitory concentrations (1/4×MICs) of the drugs. OD600 nm values are the means of three independent experiments. The control (untreated) set was included to monitor normal bacterial growth.

Fig. S2 Effect of sub-inhibitory concentrations (1/4 × MICs) of raloxifene, ezetimibe, propafenone, nefazodone, chlorprothixene, pyrvinium on the efflux inhibition of EtBr against *S. aureus* SA-1199B (*norA* over-expressed) in the presence and absence of 0.4% glucose; the positive control reserpine was included for comparison. The results presented correspond to the average of three independent assays ± SD.

Fig. S3 Effect of sub-inhibitory concentrations (1/4 × MICs) of raloxifene, ezetimibe, propafenone, nefazodone, chlorprothixene, pyrvinium on the efflux inhibition of EtBr against *S. aureus* K1758 (*norA* deletion) in the presence and absence of 0.4% glucose. The results presented correspond to the average of three independent assays ± SD.

Table S1 Binding energy (kcal/mol) of the lead compounds to the homology model of NorA efflux pump.

Compounds	Docking Score (kcal/mol)	Residues Implicated in the Interaction	MMGBSA Score (kcal/mol)
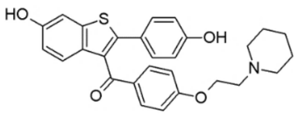<br>Raloxifene	-9.064	Hydrophobic: Ile19, Phe303, Leu218, Tyr225,<br>Hydrogen bonding : Asn340, Phe16<br>Pi-Pi stacking: Phe140<br>Polar: Gln51, Ser337, Thr336<br>Charged (Negative): Glu222	-70.08
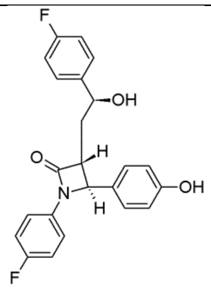<br>Ezetimibe	-7.807	Hydrogen bonding: Gly106, Gln51, Thr336, Asn340<br>Hydrophobic: Val108, Phe16, Ile19, Phe140, Ala215<br>Polar: Gln51, Ser337, Thr336<br>Charged (Positive): Arg310	-51.65
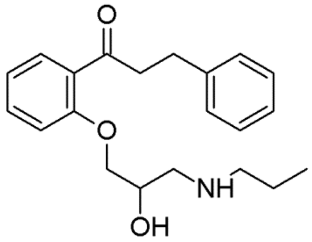<br>Propafenone	-7.848	Hydrophobic: Ile19, Val108, Leu218, Phe16, Met109, Ala215<br>Hydrogen bonding : Asn340, Thr336<br>Polar: Gln51, Ser337, Thr336<br>Charged (Positive): Arg310	-48.41
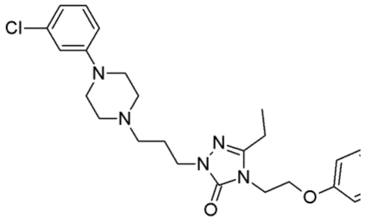<br>Nefazodone	-7.756	Hydrophobic: Ile19, Phe303, Leu218, Phe140,<br>Hydrogen bonding : Asn340<br>Polar: Gln51, Ser337, Thr336<br>Charged (Positive): Arg310	-60.78
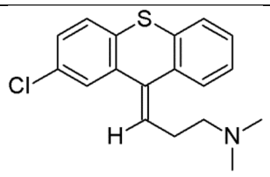<br>Chlorprothixene	-3.757	Hydrophobic: Val44, Ile19, Phe47, Ala105, Met109<br>Polar: Asn340, Ser337, Thr336, Gln51<br>Charged (Positive): Arg310	-50.18
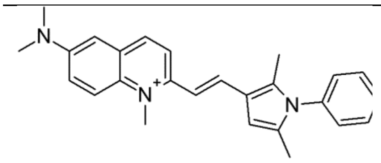<br>Pyrvinium	-4.217	Hydrophobic: Ile136, Phe16, Leu218, Met132, Tyr225, Met109<br>Pi-Pi stacking: Phe140<br>Polar: Asn340, Ser337, Thr336<br>Charged (Positive): Arg310, Lys238<br>Charged (Negative): Glu222	-45.35

Table S2 The MIC of ciprofloxacin, norfloxacin in combination with compounds for *S. aureus* SA1199 (Wild-type strain).

	Intrinsic MIC (µM)	Concentration used (µM)	MIC in the presence of compounds	
			Ciprofloxacin	Norfloxacin
			0.5	1
Raloxifene	>200	50	0.125	0.25
		25	0.25	0.5
		12.5	0.5	1
Ezetimibe	>200	50	0.25	0.25
		25	0.25	1
		12.5	0.5	1
Propafenone	>200	50	0.25	0.5
		25	0.5	0.5
		12.5	0.5	1
Nefazodone	>200	50	0.25	0.5
		25	0.5	1
		12.5	0.5	1
Chlorprothixene	>200	50	0.25	0.5
		25	0.25	0.5
		12.5	0.5	1
Pyrvinium	3.125	0.78	0.125	0.25
		0.39	0.25	0.5
		0.195	0.5	1
Reserpine	210	52	0.25	0.5
		26	0.25	0.5
		13	0.5	1

Table S3 MIC of tetracycline, and moxifloxacin for *S. aureus* XU212, and *S. aureus* SA-1199B respectively in combination with different drugs.

Compounds	*S. aureus* XU212			*S. aureus* SA-1199B		
	Intrinsic MIC (µM)	Conc. used (µM)	MIC[a] (µg/mL)	Intrinsic MIC (µM)	Conc. used (µM)	MIC[b] (µg/mL)
	-	-	256	-	-	0.25
Raloxifene	200	50	256	200	50	0.25
Ezetimibe	≥100	50	256	200	50	0.25
Propafenone	≥200	50	256	800	50	0.25
Nefazodone	≥200	50	256	200	50	0.25
Chlorprothixene	100	12.5	256	200	50	0.25
Pyrvinium	3.125	0.78	256	1.56	0.39	0.25

[a] MIC of tetracycline in the presence of compounds; [b] MIC of moxifloxacin in the presence of compounds.

Table S4 MIC of moxifloxacin for *S. aureus* clinical strains (MRSA) in combination with different drugs.

Compounds	Concentration used (µM)	MIC of moxifloxacin in the presence of compounds					
		MRSA 1	MRSA2	MRSA3	MRSA4	GMCH 831	GMCH 839
-	-	2	1	2	4	1	1
Raloxifene	50	2	1	2	4	1	1
Ezetimibe	50	2	1	2	4	1	1
Propafenone	50	2	1	2	4	1	1
Nefazodone	50	2	1	2	4	1	1
Chlorprothixene	50	2	1	2	4	1	1
Pyrvinium	0.195	2	1	2	4	1	1

Supplementary figures

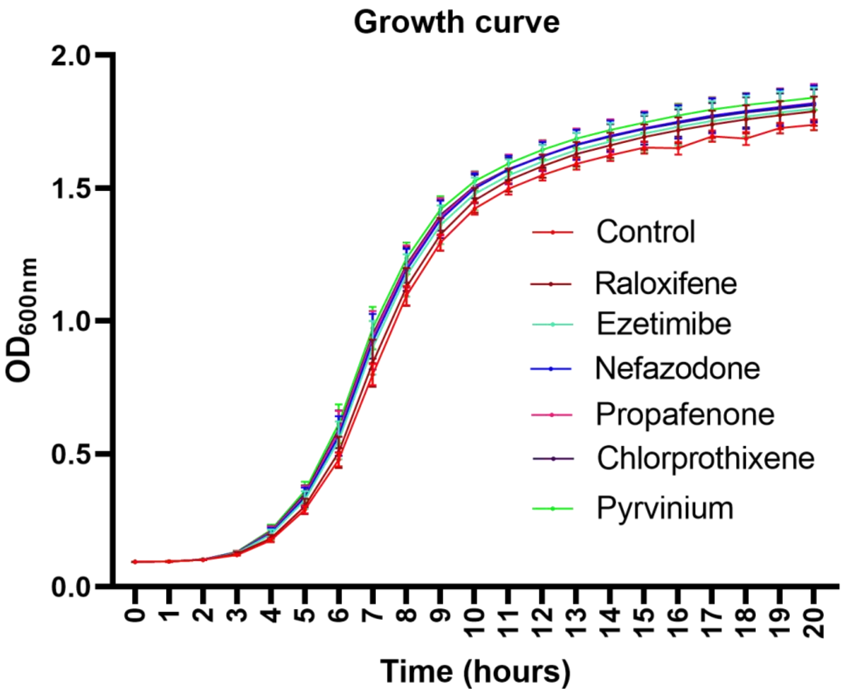

Fig. S1 Growth kinetics of *Staphylococcus aureus* SA-1199B in CA-MHB under sub-inhibitory concentrations (1/4×MICs) of the drugs. OD_{600nm} values are the means of three independent experiments. The control (untreated) set was included to monitor normal bacterial growth.

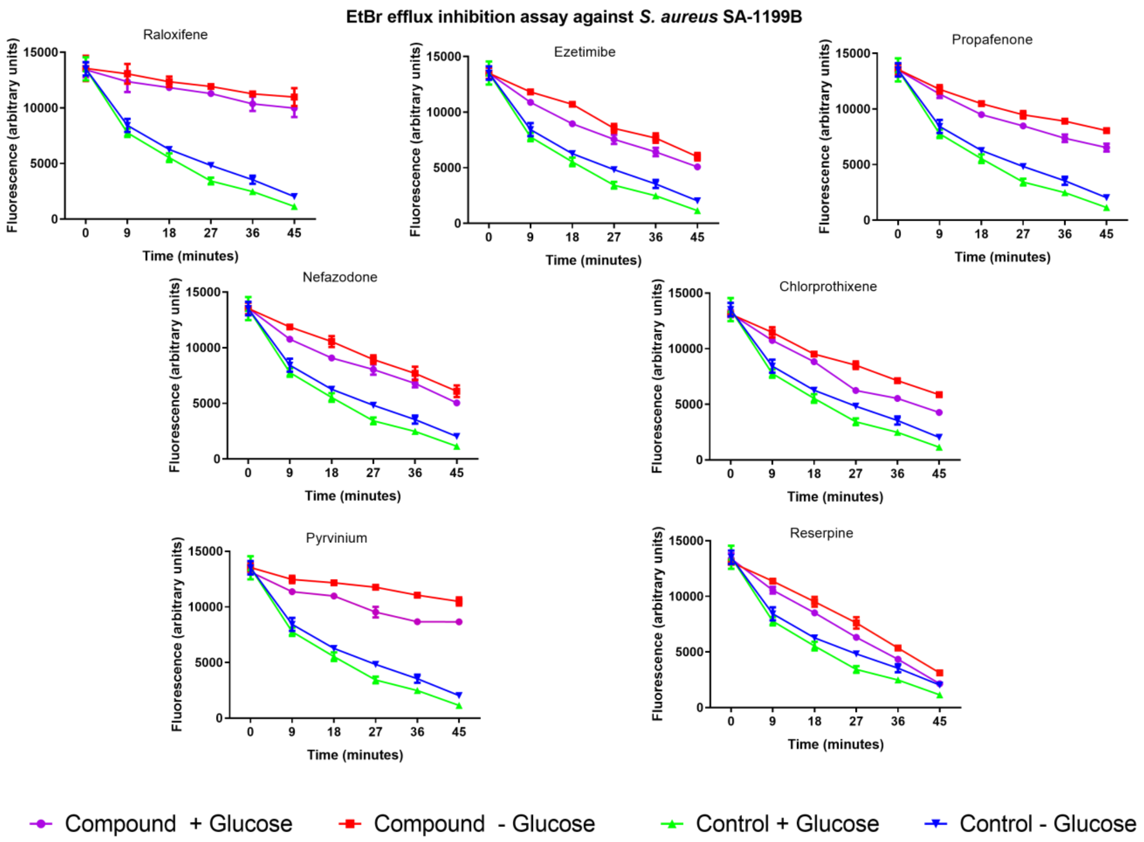

Fig. S2 Effect of sub-inhibitory concentrations (1/4 × MICs) of raloxifene, ezetimibe, propafenone, nefazodone, chlorprothixene, pyrvinium on the efflux inhibition of EtBr against *S. aureus* SA-1199B (*norA* over-expressed) in the presence and absence of 0.4% glucose; the positive control reserpine was included for comparison. The results presented correspond to the average of three independent assays ± SD.

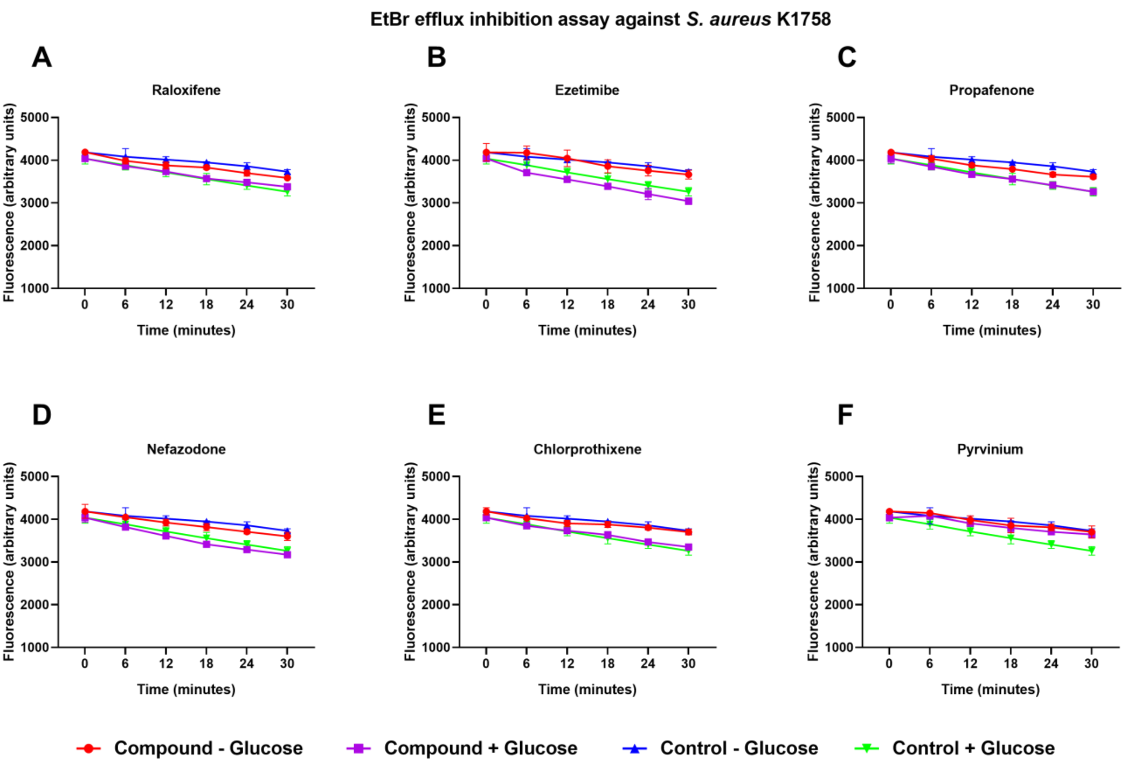

Fig. S3 Effect of sub-inhibitory concentrations (1/4 × MICs) of raloxifene, ezetimibe, propafenone, nefazodone, chlorprothixene, pyrvinium on the efflux inhibition of EtBr against *S. aureus* K1758 (*norA* deletion) in the presence and absence of 0.4% glucose. The results presented correspond to the average of three independent assays ± SD.
